# Five Visual and Olfactory Target Genes for RNAi in *Agrilus Planipennis*

**DOI:** 10.3389/fgene.2022.835324

**Published:** 2022-02-04

**Authors:** Zhizhi Fan, Zhen Zhang, Xun Zhang, Xiangbo Kong, Fu Liu, Sufang Zhang

**Affiliations:** Key Laboratory of Forest Protection of National Forestry and Grassland Administration, Research Institute of Forest Ecology, Environment and Nature Conservation, Chinese Academy of Forestry, Beijing, China

**Keywords:** RNA interference, *Agrilus planipennis*, real-time quantitative PCR, vision, olfaction

## Abstract

RNA interference (RNAi) is a widely used technique for gene function researches and recently pest controls. It had been applied in emerald ash borer (EAB *Agrilus planipennis*) larvae and adults, and achieved significant interference effects, whether by ingesting or microinjecting. Feeding in the phloem and cambial regions, the larvae of *A. planipennis* are difficult to be controlled by conventional insecticides, so adult stage is the critical stage for EAB control. However, the target genes of adult stage of *A. planipennis* need to be further screened. Here, we preliminarily screened five potential target genes of vision and olfaction for RNAi in *A. planipennis*. Three odorant binding proteins (OBPs) and three opsins, which expressed significantly different between newly emerged and sexually mature EABs (*OBP5, OBP7, OBP10, LW opsin 1* and *UV opsin 2*) or highly in sexually mature male EAB (*UV opsin 3*), were selected as targets to design primers for gene silencing. After dsRNA injection, the gene expression levels were determined by real-time quantitative PCR. We found that the expression levels of five genes were significantly down-regulated, during the 4 days after dsRNA injection. Among these genes, the expression of *LW opsin 1* was down-regulated the most, causing a reduction of 99.1% compared with the control treated with *EGFP* dsRNA, followed by *UV opsin 3* (97.4%), *UV opsin 2* (97.0%), *OBP7* (96.2%), and *OBP10* (88.7%). This study provides a basis for further RNAi-based new controlling method development of *A. planipennis* at adult stage.

## Introduction

The emerald ash borer*, Agrilus planipennis* (Coleoptera: Buprestidae) is an important native wood-boring species in Asia (Wang et al., 2010), feeding ash trees (*Fraxinus* spp.). It was not reported as a pest in Asia and eastern Russia since 2004 (Wei et al., 2004). Destruction occurred firstly in North America, and now the damage to the introduced ash trees in China is also becoming more and more serious (Wang et al., 2010). The larvae of *A. planipennis* feed in the phloem and cambial regions, creating S-shaped galleries that disrupt the tree’s transport of nutrients and water, causing rapid death after a series of attack about 2–4 years (Liu et al., 2003). It is difficult to control the larvae using conventional insecticides, so adult stage is the critical stage for EAB control (Fan et al., 2021). Chemical control is effective, but it also injures the natural enemies of *A. planipennis* and is economically and ecologically unsustainable (Davidson and Rieske, 2016). Based on this situation, new efficient and sustainable biological control technologies are urgently needed.

RNA interference (RNAi) is a widely used technique for gene function researches and recently pest controls (Zhao et al., 2015). The entomological researchers have been trying to use RNAi technology in different species of insects and resulted in some valuable evidences (Zhang et al., 2017; Niu et al., 2018; Zotti et al., 2018). Feeding insects with plants which express dsRNA, the expression of target genes were knockdown successfully in *Diabrotica virgifera* (Baum et al., 2007), *Helicoverpa armigera* (Mao et al., 2007), *Bemisia tabaci* (Tian et al., 2009), *Myzus persicae* (Pitino et al., 2011), *Leptinotarsa decemlineata* (Jiang et al., 2015) and *Manduca sexta* (Poreddy et al., 2017)*.* Due to the high specificity and efficiency of RNAi, more and more research groups tried to develop new pest control methods based on this technology and the latest research results showed that inhibiting the expression of important genes in the growth and development process of insects could cause growth and development disorders or death of insects. Many relevant target genes were screened, such as the inhibitor of apoptosis (*IAP*), vacuolar sorting protein *SNF7* (*SNF7*), and snakeskin (*SSK*) of *Anoplophora glabripennis* (Dhandapani et al., 2020); the chitinase (*Sf-CHI*), chitin synthase B (*Sf-CHSB*), sugar transporter SWEET1 (*Sf-ST*), and hemolin (*Sf-HEM*) of *Spodoptera frugiperda* (Wan et al., 2021); the leucine-rich repeat-containing (*LGR2*) of *Hyphantria cunea* (Sun et al., 2021) and so on. All of above is the strong evidence that RNAi can be used as a new method for pest control.

It is necessary that an organism possesses the RNAi machinery to silence a target gene (Zhao et al., 2015). Dicer enzymes, argonaute endonucleases, and dsRNA-binding proteins are three core components of RNAi machinery (Swevers et al., 2013). These three components have been identified in the genome sequence of *A. planipennis* (Zhao et al., 2015)*.* Several studies have also successfully applied RNAi to larvae and adults *A. planipennis*, achieving significant interference effects, whether by ingesting or microinjecting (Rodrigues et al., 2017; 2018). Also, feeding neonates with dsRNAs that was specifically expressed in *E. coli* successfully silenced related genes and caused mortality (Leelesh *and* Rieske, 2020). Pampolini *et al.* (2020) found that adult EABs experienced a significant knockdown of the *shi* gene and beetles mortality, after feeding with tropical ash leaves which were treated with *dsSHI* through petiole absorption. The above studies indicate that *A. planipennis* is sensitive to dsRNA, which can be triggered by exogenous dsRNA molecules to knock down the target genes. However, effective adult RNAi target genes is lack in *A. planipennis* since now.

Olfaction (Bartelt et al., 2007; Lelito et al., 2009; Mccullough et al., 2009; Pureswaran and Poland, 2009) plays an important role in EAB host location and mating. These behaviors depend on insects’ perception of various chemical signals (Wang et al., 2020). As the main peripheral olfactory proteins, odorant binding proteins (OBPs) play a key role in insect olfactory system (Wang et al., 2020). Therefore, it is feasible to screen OBPs as targets for RNAi of *A. planipennis*, and some progress have been made in the identification of EAB OBPs. Five OBPs and one chemosensory proteins (CSPs) were identified from 16,000 expression sequence tags generated from the antennae and legs of male and female EABs in 2012 (Qazi et al., 2012). Based on the antennal transcriptome, identified nine OBPs in *A. planipennis.* Andersson *et al* (2019) identified twelve OBPs based on the newly analyzed genome of *A. planipennis*. While we found six new OBPs in our transcriptome assembly (Shen et al., 2021), further improving the data integrity of olfactory related genes identification of EAB.

Vision is important during food searching, mating, and predators avoiding of insect (Endler and Mappes, 2004; Lelito et al., 2007; Lelito et al., 2008; Everett et al., 2012), and it is supported by visual pigment molecules which contain a chromophore and a seven transmembrane opsin (Shichida and Imai, 1998). The spectral sensitivity of photoreceptors is determined by the amino acid sequences of opsin and chromophore (Terakita, 2005). As an important protein in the complex visual system, opsin is an important photosensitive substance widely existing in insects (Porter et al., 2012; Roberto et al., 2016). So opsins are suitable RNAi targets for insect control and EAB opsin researches have made some progress at present. Lord *et al* (2016) identified two UV opsins, two LW opsins copies and a partial third LWS opsin copy in the male EAB, and we identified three UV opsins, one UV opsin-like gene, two Green opsins, and two LW opsins in our transcriptome assembly (Shen et al., 2021). Identification of important visual and olfactory genes and the application of RNAi in *A. planipennis* makes target genes explore feasible in adult EABs. In this study, we designed primers of the identified olfactory and visual genes to synthesize dsRNA for RNAi, the interference effects of them were tested by quantitative real-time PCR. Finally, we preliminarily screened five potential target genes of visual and olfactory for RNAi in adult *A. planipennis.* Our results provide a basis for further olfactory and visual mechanism researches of *A. planipennis*, and the primers may be used in RNAi-based control of this pest.

## Materials and Methods

### Insects

*A. planipennis* were collected from the Tongzhou district of Beijing and Chengde, Hebei, China during May and June 2021. Adults were reared in the ventilated plastic boxes with evergreen ash (*Fraxinus uhdei*) foliage in a vial of water for feeding (Zhao et al., 2015), maintained in the laboratory at 26°C ± 2°C, with 25% ± 5% relative humidity and under a 14:10 h light/dark photoperiod. Collected adults were reared overnight in the laboratory, and then the healthy beetles were selected for the injection experiment.

### Double-Stranded RNA Synthesis

Candidate genes were selected based on our previous work (Shen et al., 2021), the selected candidates were expressed significantly different between newly emerged and sexually mature *A. planipennis* (*OBP5*, *OBP7*, *OBP10*, *LW opsin 1* and *UV opsin 2*) or highly expressed in sexually mature *A. planipennis* (*UV opsin 3*). The control consisted injections of *dsEGFP* (dsRNA against green fluorescent protein), H_2_O and nontreated. Double-stranded RNA (dsRNA) was synthesized by using T7 RNA polymerase and gene-specific primers. The primers were designed with a T7 polymerase promoter sequence (TAA​TAC​GAC​TCA​CTA​TAG​G) at the 5’ end (Supplementary Table S1) to generate PCR templates for *in vitro* transcription of dsRNA. PCR conditions were 94°C for 2 min, followed by 35 cycles of 94°C for 30 s, 55°C for 30 s and 72°C for 1 min, finishing with an extension step at 72°C for 10 min. The PCR templates were purified using a universal DNA Purification Kit (Tiangen, Beijing, China). After purification, dsRNA was synthesized and purified using the T7 RiboMAX™ Express RNAi System (Promega, United States) following instructions provided. The synthesized dsRNA was then quantified using NanoDrop ND-2000 (Thermo Scientific, Wilmington, DE, United States), and its quality was examined by agarose gel electrophoresis. The final concentration of dsRNA was diluted to 2 μg/μl.

### Microinjection of dsRNA

Healthy adults *A. planipennis* of similar size were selected and placed in Petri dishes. 1.5 µl dsRNA was injected into the internode membrane between the first and second abdominal segments, using the microinjector (Hamilton, United States). The injected adults were reared again in the plastic boxes at 26°C ± 2°C in the laboratory. Samples were collected at 6 hours, 1, 2, 3 and 4 days after injection and immediately frozen in liquid nitrogen and stored at -80°C. Four biological replications were prepared for each sample.

### Total RNA Isolation and First-Strand cDNA Synthesis

Trizol reagent (Invitrogen, Carlsbad, CA, United States) was used to extract the total RNA of a single dsRNA-injected or control beetles following the manufacturer’s instructions (Zhang et al., 2014). The concentration of RNA was measured by NanoDrop ND-2000 and the quality was assessed by agarose gel electrophoresis. The final concentration of total RNA was diluted to 1 μg/μl.

The first strand cDNA was synthesized using a GoScript™ Reverse Transcription System (Promega, United States) with 4 µg total RNA following the provided instructions. The concentration of cDNA was measured by NanoDrop ND-2000 and the quality was assessed by agarose gel electrophoresis. The final concentration of cDNA was diluted to 40 ng/μl before gene expression quantification.

### Quantitative Real-Time PCR

Resulted cDNA above was used as template for qPCR. Primer Premier 5.0 software was used to designed primer pairs (Supplementary Table S2), and the parameters were set as 20bp±1bp in length, melting temperature (Tm) of 50–60°C and a product size of 150–250 bp (Priya et al., 2012). 20µl PCR system were mixed following the SuperReal PreMix Plus (Tiangen, Beijing, China) introductions, contained 10 µl 2×SuperReal PreMix Plus, 0.6 µl of each primer (10 mM), 1 µl cDNA and 7.8 µl RNase-free ddH_2_O. Reaction protocols were one cycle at 95°C (3 min), followed by 40 cycles of denaturation at 95 C (5s), annealing and extension at 60 C for 30 s, running in CFX96 Touch Real-Time PCR Detection System (Bio-Rad, Hercules, CA). A melting curve was generated from 65 to 95°C after each qPCR reaction, confirming the single peak and there was no primer-dimer or non-specific product. The *TEF-1α* (Priya et al., 2012) was selected as the internal control and the relative expression level of each gene was calculated by the 2^−ΔΔCT^ method. Each sample had four biological replicates and three technical replicates.

### Statistical Analysis

Statistical tests were performed using GraphPad Prism. We used ANOVA/LSD method to analyze differences among different treatments. The level of significance of difference was set at *p* < 0.05 and a, b, c, d were used to indicate significant differences.

## Results

### dsRNA of *A. planipennis* Synthesized *in vitro*

DsRNA of each gene, examined by agarose gel electrophoresis, showed integrity and the expected size (Supplementary Figure S1). The amplicon size was of 678 bp for *dsEGFP*, 314 bp for *dsOBP10*, 311 bp for *dsOBP7*, 350 bp for *dsOBP5*, 321 bp for *dsLW1*, 440 bp for *dsUV2*, 287 bp for *dsUV3.*

### Specificity Detection of qPCR Primers

PCR amplification of each pair of qPCR primers resulted single amplicon with desired size on 1.0% agarose gel (Supplementary Figure S2). The fluorescence PCR fusion curves indicate that all genes are single-peak and without heterobands, which prove the specificity of the designed primers (Supplementary Figure S3).

### Analysis of RNAi Effect

To assess the RNAi effect on candidate genes, expression analysis were performed at 6 hours, 1, 2, 3 and 4 days after dsRNA injection, using *TEF-1α* as internal control. DsRNA injections of both olfactory (except for *OBP5*) and visual genes resulted in some degree of down-regulation comparing with expression in control beetles (*EGFP* dsRNA controls).

**FIGURE 1:**
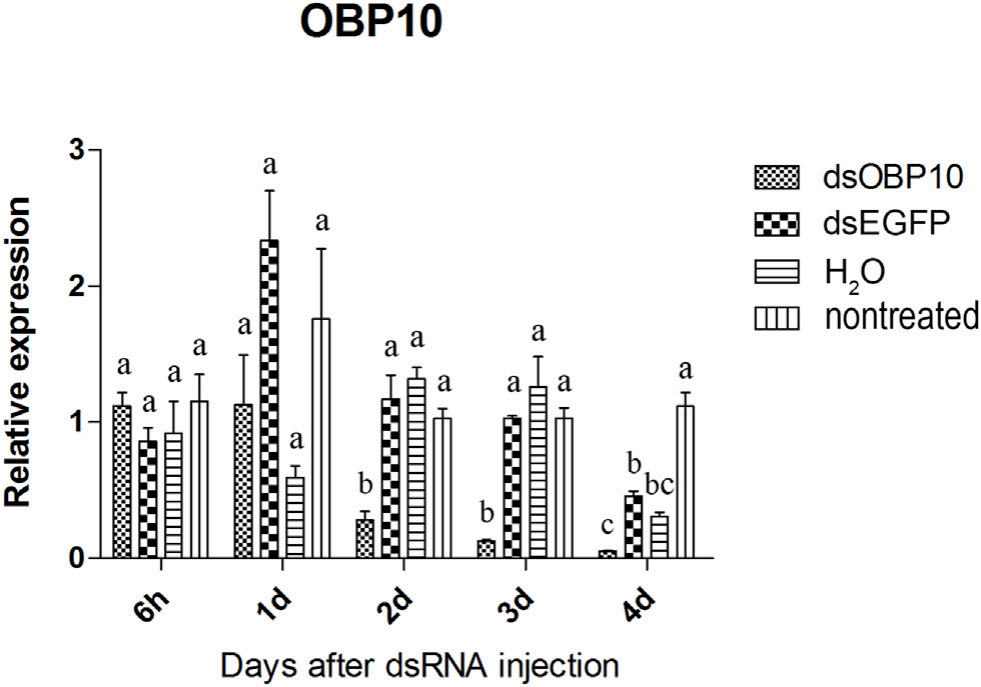
Relative expression of *OBP10* in EAB adults after dsRNA injection. Relative expression of *OBP10* by RT-qPCR assay was evaluated 6 hours, 1, 2, 3 and 4 days after injecting 3 µg dsRNA. *TEF-1α* was used as a reference gene for normalization. Means ± SE (n = 4) are shown. Statistical analyses were performed using ANOVA/LSD method at *p* < 0.05 for comparison among treatments. Statistical differences are shown by different letters between different treatments at the same time point.

**FIGURE 2:**
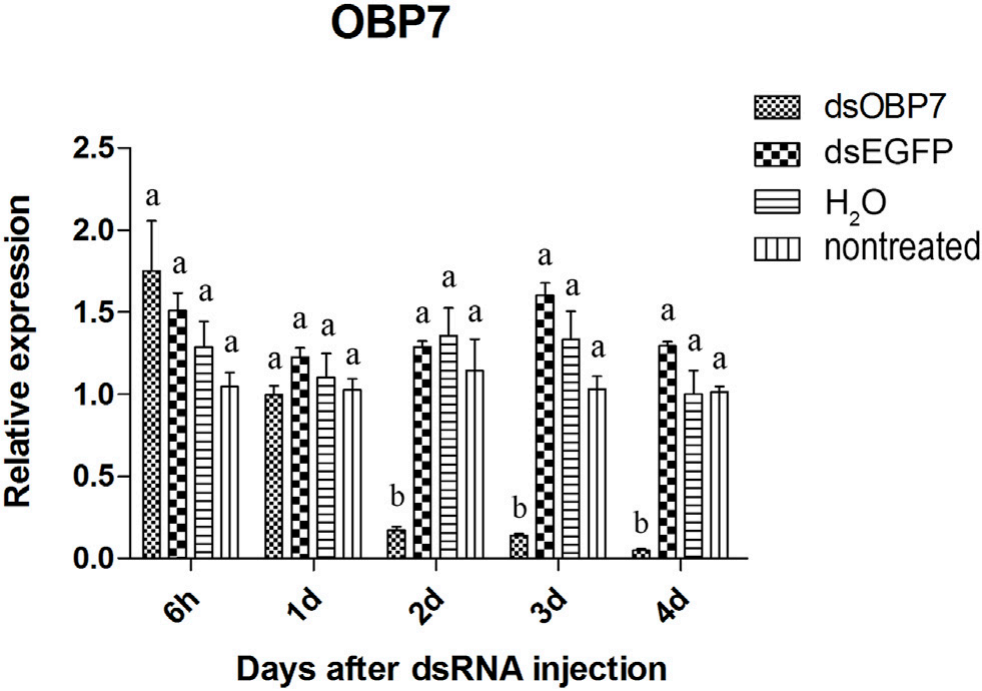
Relative expression of *OBP7* in EAB adults after dsRNA injection. Relative expression of *OBP7* by RT-qPCR assay was evaluated 6h, 1, 2, 3 and 4 days after injecting 3 µg dsRNA. *TEF-1α* was used as a reference gene for normalization. Means ± SE (n = 4) are shown. Statistical analyses were performed using ANOVA/LSD method at *p* < 0.05 for comparison among treatments. Statistical differences are shown by different letters between different treatments at the same time point.

**FIGURE 3:**
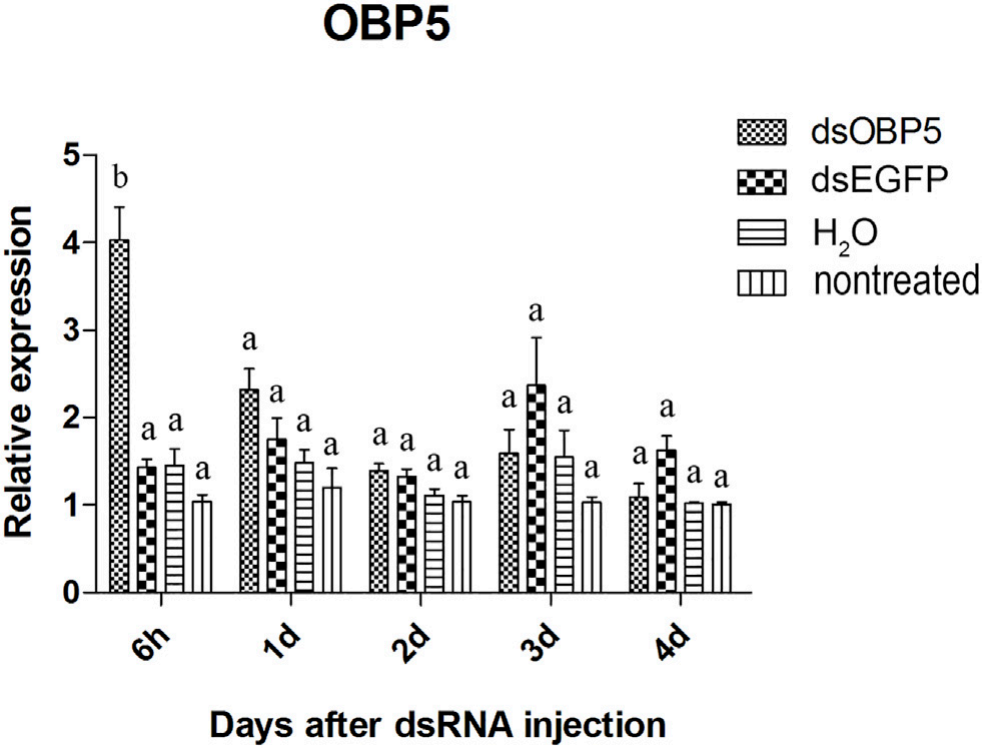
Relative expression of *OBP5* in EAB adults after dsRNA injection. Relative expression of *OBP5* by RT-qPCR assay was evaluated 6h, 1, 2, 3 and 4 days after injecting 3 µg dsRNA. *TEF-1α* was used as a reference gene for normalization. Means ± SE (n = 4) are shown. Statistical analyses were performed using ANOVA/LSD method at *p* < 0.05 for comparison among treatments. Statistical differences are shown by different letters between different treatments at the same time point.

Two of the three olfactory genes were significantly down-regulated after dsRNA injection. Two days after injection, the relative expression level of *OBP10* significantly decreased. The lowest relative expression level of *OBP10* mRNA appeared at 4 days post-injection and the expression level was reduced by 88.7%, compared to *dsEGFP*-treated beetles (Figure 1). Similar degree of expression suppression happened in *dsOBP7*-treated beetles. At 4 days post-injection, injection of *dsOBP7* in adult beetles resulted in significant silencing of nearly 96.2% when compared with expression in *dsEGFP*-treated beetles (Figure 2). At 6 h post-injection, a significantly elevated expression was observed for *OBP5* comparing to control *EGFP* dsRNA. From day 1, the expression level of OBP5 returned to normal and continued until day 4, with no significant difference comparing with the expression level in *dsEGFP*-treated beetles (Figure 3).

**FIGURE 4:**
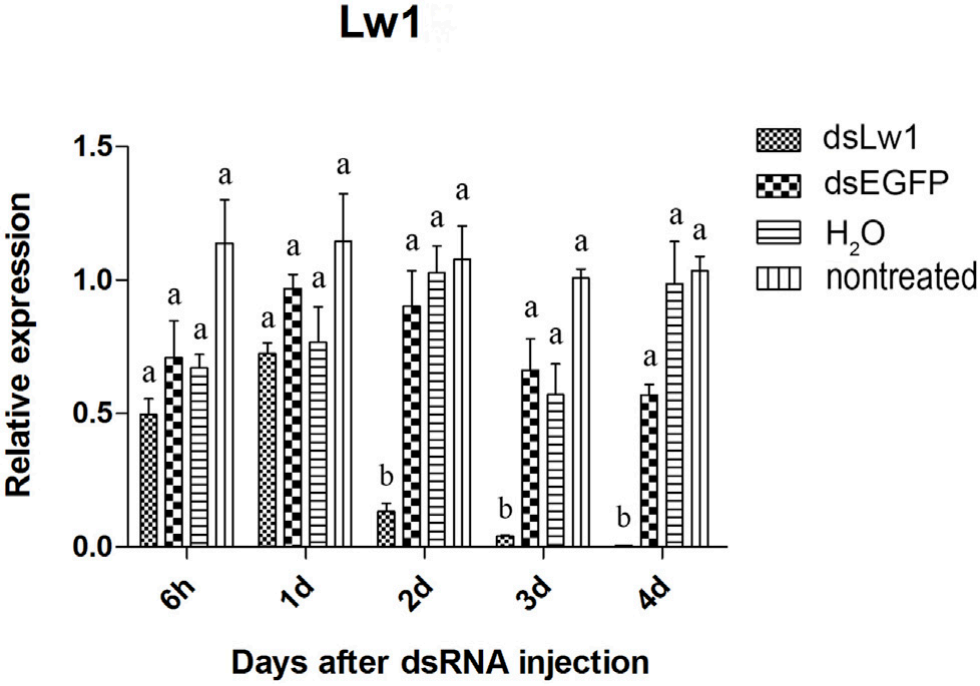
Relative expression of *LW opsin 1* in EAB adults after dsRNA injection. Relative expression of *LW opsin 1* by RT-qPCR assay was evaluated 6 hours, 1, 2, 3 and 4 days after injecting 3 µg dsRNA. *TEF-1α* was used as a reference gene for normalization. Means ± SE (n = 4) are shown. Statistical analyses were performed using ANOVA/LSD method at *p* < 0.05 for comparison among treatments. Statistical differences are shown by different letters between different treatments at the same time point.

**FIGURE 5:**
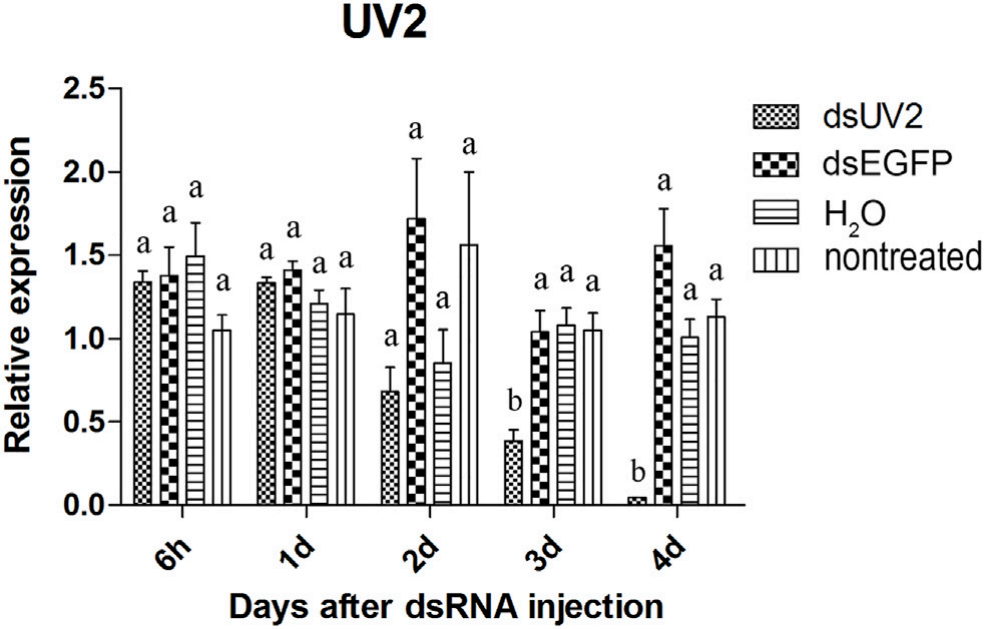
Relative expression *UV opsin 2* in EAB adults after dsRNA injection. Relative expression of *UV opsin 2* by RT-qPCR assay was evaluated 6 hours, 1, 2, 3 and 4 days after injecting 3 µg dsRNA. *TEF-1α* was used as a reference gene for normalization. Means ± SE (n = 4) are shown. Statistical analyses were performed using ANOVA/LSD method at *p* < 0.05 for comparison among treatments. Statistical differences are shown by different letters between different treatments at the same time point.

**FIGURE 6:**
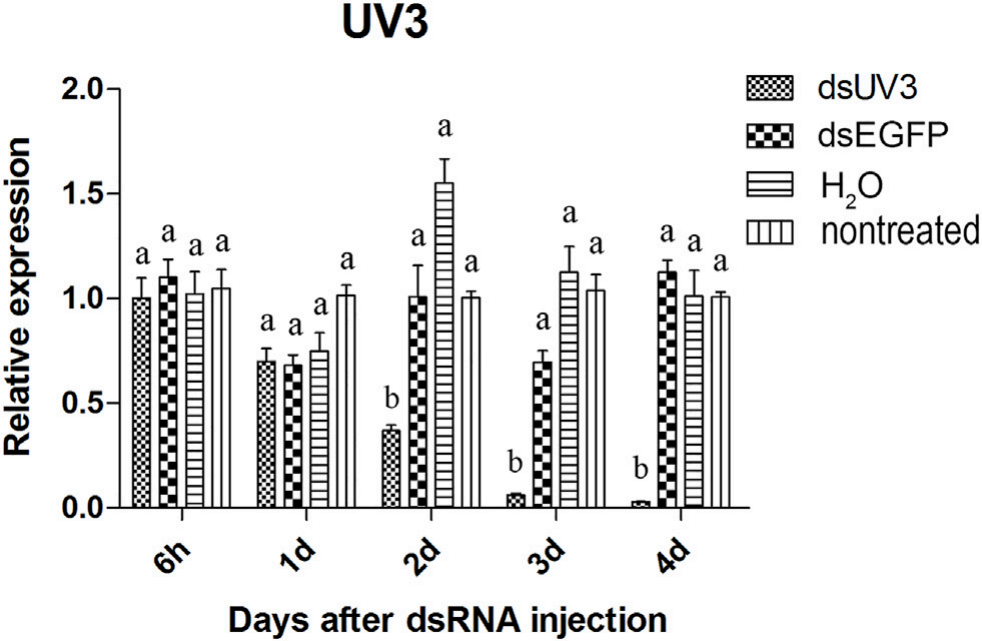
Relative expression of *UV opsin 3* in EAB adults after dsRNA injection. Relative expression of *UV opsin 3* by RT-qPCR assay was evaluated 6 hours, 1, 2, 3 and 4 days after injecting 3 µg dsRNA. *TEF-1α* was used as a reference gene for normalization. Means ± SE (n = 4) are shown. Statistical analyses were performed using ANOVA/LSD method at *p* < 0.05 for comparison among treatments. Statistical differences are shown by different letters between different treatments at the same time point.

The visual genes have slightly higher knockdown rates than the olfactory genes. The expression of these genes were not significantly reduced at 6 hours and 1 day after injection of the dsRNAs comparing to *dsEGFP*-treated beetles (Figures 4–6). Two days after injection, significant gene knockdown was observed in beetles injected with *LW opsin 1* and *UV opsin 3* dsRNA (*dsLW1* and *dsUV3*) (Figures 4, 6) comparing to *dsEGFP*-treated beetles. Among these genes, the expression of *dsLW1*-treated beetles was down-regulated the most, causing a reduction of 99.1% comparing with the *dsEGFP*-treated beetles (Figure 4). The expression of *UV2* did not reduce significantly until the third day after injection, and fell by 97.0% at 4 days post-injection (Figure 5). Compared with *EGFP* controls, the expression of *UV3* was significantly reduced by more than a half at 2 days post-injection, reaching 97.4% on day 4 (Figure 6).

## Discussion

The emerald ash borer is an important pest of *Fraxinus* spp. that has caused huge economic losses in China. Since most of its life cycle is hidden under the bark of host and only appears outside the bark about 1 month during the adult stage, it is difficult to be controlled (Wang et al., 2010). Currently, physical (Crook et al., 2009; Poland and Mccullough, 2014; Ryall et al., 2015), chemical (Nicole et al., 2010; Mccullough et al., 2016; Poland et al., 2016) and biological (Duan et al., 2020) methods are mainly used to control *A. planipennis.* All these methods are useful, but there are still some shortcomings. As a new molecular biological technology, RNAi has been applied in some insects of coleoptera (Ke et al., 2018; Haller et al., 2019; Pinheiro et al., 2020). In this study, five effective primers of visual and olfactory target genes for RNAi of EAB were designed, providing a basis for RNAi-based new controlling method development of *A. planipennis* at adult stage.

The results of qPCR showed that the interference effect of the visual genes in EAB were very obvious. Four days after *dsLW1* treatment resulted a knockdown of nearly 100% mRNA (Figure 4). Similar degrees of gene suppression in other two visual genes (Figures 5, 6) and *OBP7* (Figure 2) were observed. The knockdown rate of *OBP10* was slightly lower than other genes, but also reached 88.7% (Figure 1). The results indicated that the expression levels of candidate genes after RNAi were significantly down-regulated and the knockdown rates were high, which means we preliminarily screened five potential target genes of vision and olfaction for RNAi in *A. planipennis* at adult stage.

The suppressed expression of a specific OBP often show altered behavioral responses to more than one odorants (Swarup et al., 2011). For example, the sensitivity to the EO (essential oil) of *Tribolium castaneum* had decreased after *TcOBPC11* knockdown and caused a higher mortality, which suggested that this OBP gene was related to defense of the beetles against EO (Zhang et al., 2020). Similarly, responses to specific odors are often the result of multiple OBP expressions (Swarup et al., 2011). When *BodoOBP1* and *BodoOBP2* in males of *Bradysia odoriphaga* were knocked down, their ability to search females was significantly reduced comparing to control (Bowen et al., 2019). RNAi of *OBP7* and *OBP10* may also lead to significant physiological changes in EABs, and this deserved further investigation in our future work.

Opsin silencing related work in insects are relatively fewer (French et al., 2015). Injecting the dsRNAs of *opsin-Long Wave (opLW)* into compound eyes of *Gryllus bimaculatus* led to photic entrainability losing, and suggested that *opLW* is the major photoreceptor molecule for photic entrainment of the cricket’s circadian clock (Komada et al., 2015). A few studies related to the interference of insect opsins showing that, except for the quantity of dsRNA injected, the gene silencing effect also seems to depend on the circadian state of the visual system (Leboulle et al., 2013). We need to take into account of this factor in the application research of visual interference in the future. Several work that evaluating colors traps for *A. planipennis* have yielded similar results that *A. planipennis* is sensitive to purple and green traps (Francese et al., 2010; Poland and Mccullough, 2014; Petrice and Haack, 2015). Whether opsins knockdown can affect the color sensitivity of *A. planipennis* is unknown, and no related research on coleoptera pests were performed as we know. However, the down-regulated visual gene reduced the preference response of pests to green, which was verified in the interference experiment of *Bactrocera minax* (Wang et al., 2018). It will be our main research content in the future to verify the effect of visual gene interference using colored sticky cards.

Currently, most of the RNAi experiments on EAB were applied on the larval stage, focusing on growth and development genes. Feeding *IAP* or *COP* (COPI coatomer, *β* subunit) dsRNA to neonate EAB larvae could silence their target genes and caused mortality (Rodrigues et al., 2017). Rodrigues *et al* also screened two efficient target genes, *hsp* (heat shock 70-kDa protein cognate 3) and *shi* (shibire), both in larvae and adults. The knockdown of these two gene could cause up to 90% mortality (Rodrigues et al., 2018). However, RNAi targets suitable for adult *A. planipennis* are still rare now*.* Behavioral experiments on vision and olfaction in *A. planipennis* proved their importance at adult stage (Lelito et al., 2008; Mccullough et al., 2009; Pureswaran and Poland, 2009). Thus, we screened five RNAi target genes aiming at these two sensory system of adult EABs in this work. Although selection of appropriate target genes is key to successful gene silencing (Leelesh and Rieske, 2020), it is only the first step for further applications, and there is still a lot of work to be done.

In the future, we will conduct physiological and behavioral experiments to verify the interference effect of the preliminarily screened genes. Gas chromatographic-electroantennographic detection (GC-EAD) will be used to measure the effects of gene interference by comparing the responses of the treatment and control groups to odors (Ngumbi et al., 2009). The visual ability of the beetles after interference will be measured by colored sticky cards (green or purple) (Francese et al., 2013).

## Data Availability

The original contributions presented in the study are included in the article/Supplementary Material, further inquiries can be directed to the corresponding author.

## Supplementary Material

Supplementary Table S1 Primers used for dsRNA synthesis.

Supplementary Table S2 Primers used for qPCR.

Supplementary Figure S1 Integrity of dsRNAs of candidate genes and dsEGFP synthesized in vitro by agarose gel analysis. M: DNA 2K marker; 1: dsEGFP; 2: dsOBP10; 3: dsOBP7; 4: dsOBP5; 5: dsLW1; 6: dsUV2; 7: dsUV3.

Supplementary Figure S2 Agarose gel analysis of PCR products of candidate genes and reference gene (TEF-1α). M: DNA 2K marker; 1: *OBP10*; 2: *OBP7*; 3: *OBP5*; 4: *LW opsin 1*; 5: *UV opsin 2*; 6: *UV opsin 3*; 7: *TEF-1α*.

Supplementary Figure S3 Dissociation curves of candidate genes during qPCR reaction.

Supplementary Figure S4 Original gel of dsRNAs of candidate genes and dsEGFP synthesized in vitro.

Supplementary Figure S5 Original gel of PCR products of candidate genes and reference gene (TEF-1α).
