# Supplementary figures and images for: Five Visual and Olfactory Target Genes for RNAi in Agrilus Planipennis
Source: Front Genet. 2022 Feb 4;13:835324. doi: 10.3389/fgene.2022.835324 (PMC8855093; doi:10.3389/fgene.2022.835324)

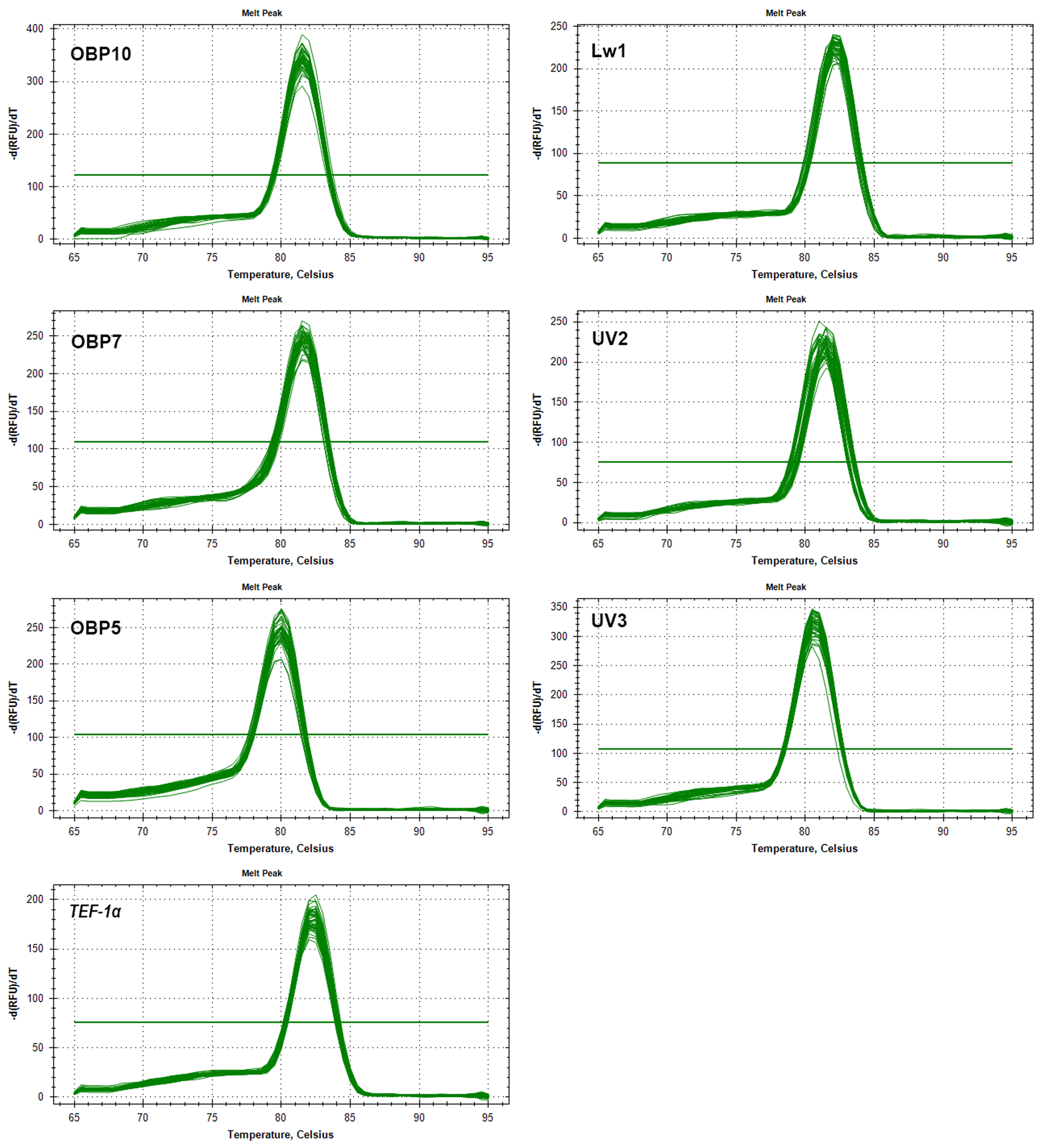

Supplement: Supplementary file 1 [file Image3.JPEG]

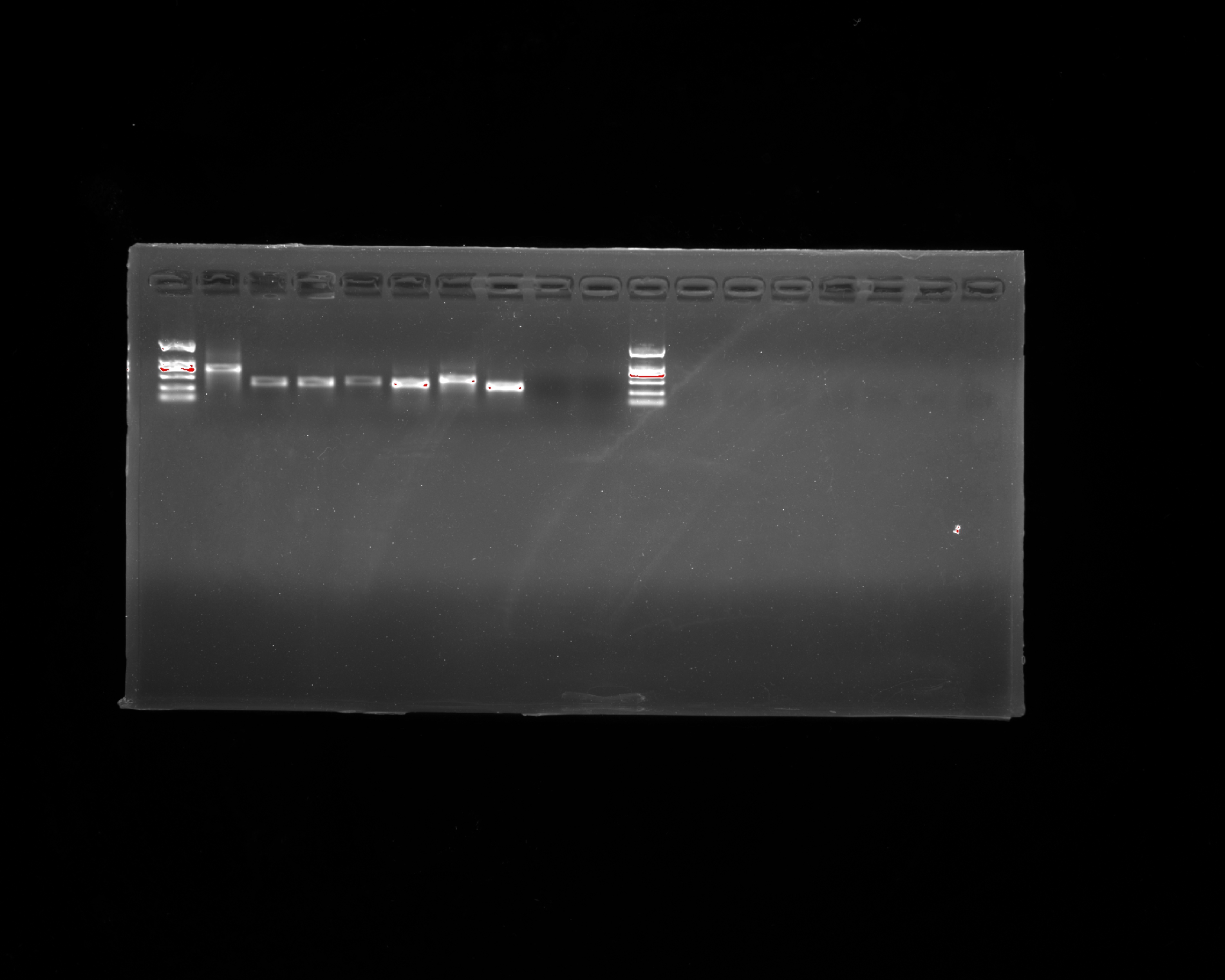

Supplement: Supplementary file 2 [file Image4.JPEG]

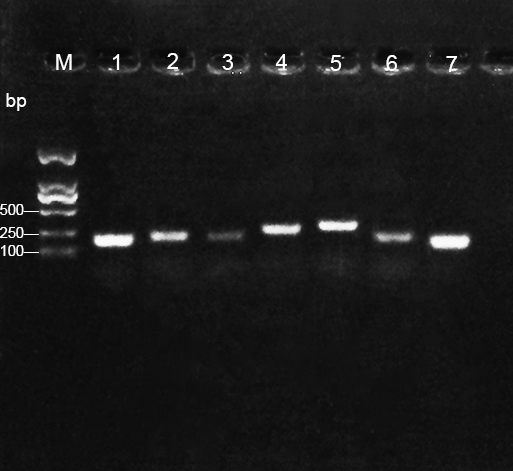

Supplement: Supplementary file 3 [file Image2.TIF]

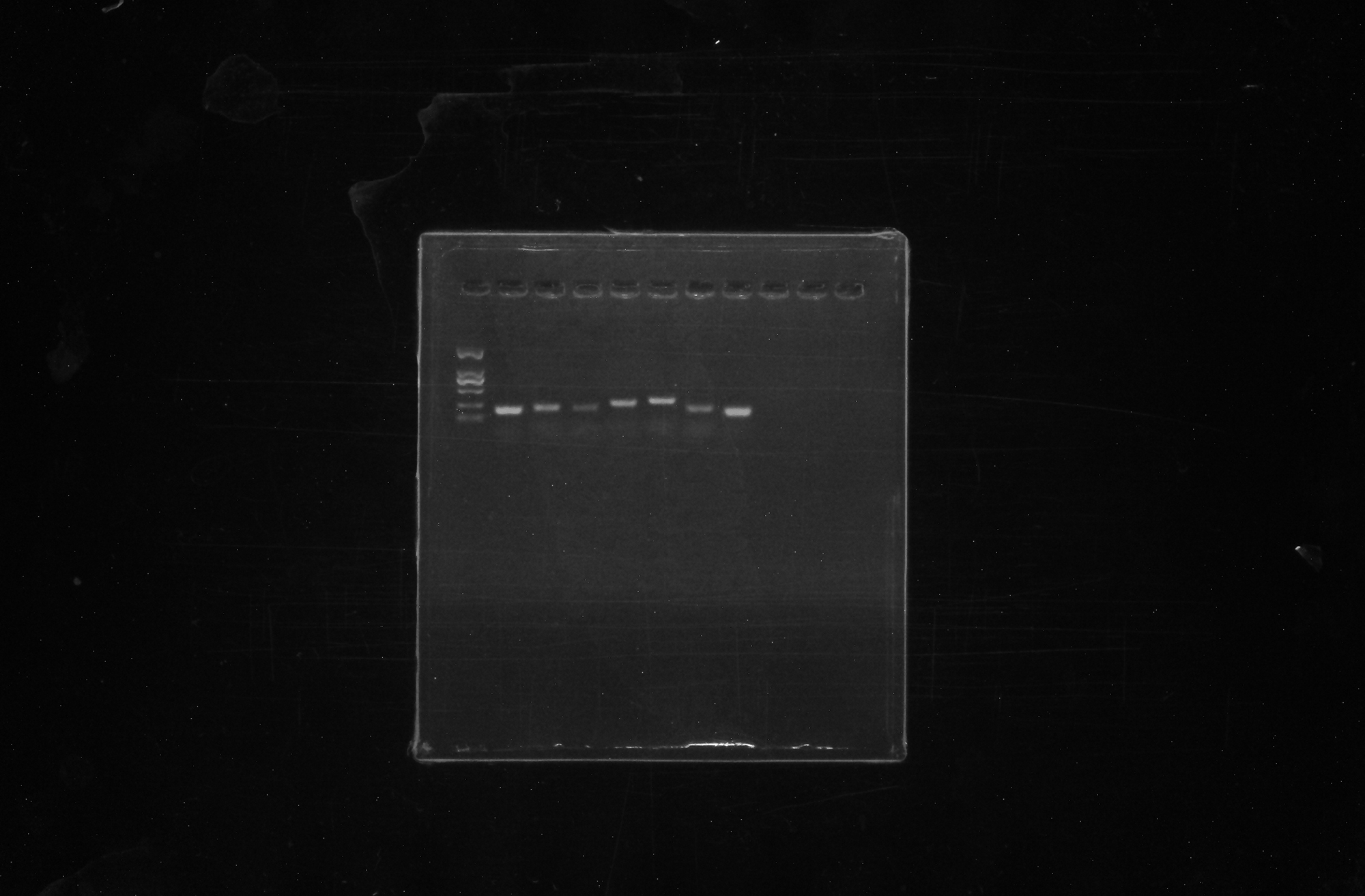

Supplement: Supplementary file 4 [file Image5.JPEG]

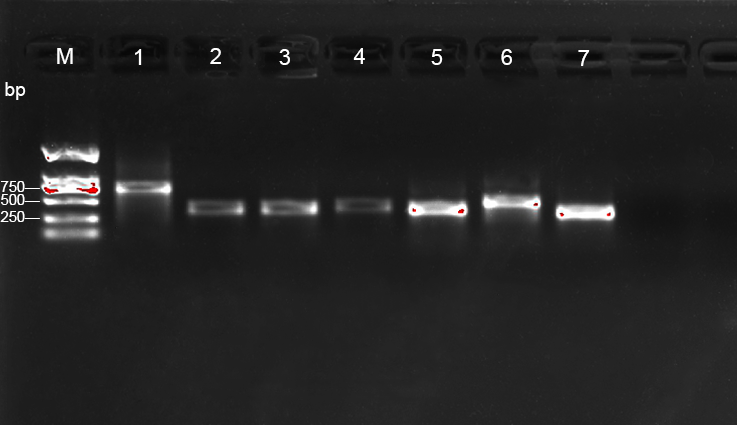

Supplement: Supplementary file 5 [file Image1.TIF]
