# Supplementary material for: Five Visual and Olfactory Target Genes for RNAi in Agrilus Planipennis
Source: Front Genet. 2022 Feb 4;13:835324. doi: 10.3389/fgene.2022.835324 (PMC8855093; doi:10.3389/fgene.2022.835324)
Supplement: Supplementary file 6 [file Table1.DOC]

**Table S1 Primers used for dsRNA synthesis**

| Genes | Sequence (5'—3') |
| --- | --- |
| *AplaOBP10-F* | TAATACGACTCACTATAGGTTTTGGACTATTTGCTAGTGT |
| *AplaOBP10-R* | TAATACGACTCACTATAGGTCGTATTTGTCTTTTCCTTTT |
| *AplaOBP7-F* | TAATACGACTCACTATAGGACAGTTCAGGCGACATTG |
| *AplaOBP7-R* | TAATACGACTCACTATAGGTTCTGCCGATTCCTTTT |
| *AplaOBP5-F* | TAATACGACTCACTATAGGCAGTTGCTCCTCTCATTTC |
| *AplaOBP5-R* | TAATACGACTCACTATAGGTTTTTTCCATCGTTGTTCT |
| *LWopsin1-F* | TAATACGACTCACTATAGGCACCTACATCTGGGCATACA |
| *LWopsin1-R* | TAATACGACTCACTATAGGCTTGGCGAATAAAGAACTCC |
| *UVopsin2-F* | TAATACGACTCACTATAGGTCCAAAAGCGGCAATAACACC |
| *UVopsin2-R* | TAATACGACTCACTATAGGCTGGCTCAAGTACCCCGAACC |
| *UVopsin3-F* | TAATACGACTCACTATAGGGGACCGTAGTTAATTCACCC |
| *UVopsin3-R* | TAATACGACTCACTATAGGACATCATCAAAAAATCGCAG |
| *EGFP-F* | TAATACGACTCACTATAGGTGAGCAAGGGCGAGGAG |
| *EGFP-R* | TAATACGACTCACTATAGGCGGCGGTCACGAACTCCAG |

Note: The underline shows the T7 promoter sequence.
