# Supplementary material for: Five Visual and Olfactory Target Genes for RNAi in Agrilus Planipennis
Source: Front Genet. 2022 Feb 4;13:835324. doi: 10.3389/fgene.2022.835324 (PMC8855093; doi:10.3389/fgene.2022.835324)
Supplement: Supplementary file 7 [file Table2.DOC]

**Table S2 Primers used for qPCR.**

| Genes | Sequence (5'—3') |
| --- | --- |
| *AplaOBP10-qF* | CGTCGTTGACGAAGAAAGC |
| *AplaOBP10-qR* | AGTGCGAATGCTGGTGGTT |
| *AplaOBP7-qF* | TGTAATTGCCACAGTTCAG |
| *AplaOBP7-qR* | TCGGGTGTAATAAATCCAG |
| *AplaOBP5-qF* | TACTTTGGGTCGTCTTCTG |
| *AplaOBP5-qR* | TTTTTTCCATCGTTGTTCT |
| *LWopsin1-qF* | TCAGCACCACCAAACACTTA |
| *LWopsin1-qR* | CGCTTAGACCCTTTACGATA |
| *UVopsin2-qF* | TCCGCTGATTGATTCGCTTGG |
| *UVopsin2-qR* | TGGGTGTTATTGCCGCTTTTG |
| *UVopsin3-qF* | ATGTTCGTTGCAGTTCTGTT |
| *UVopsin3-qR* | ATTGGTTTTGTTGCTGTTGA |
| *ApEF1-α F* | CATTGAAACCTACGTTGTCGC |
| *ApEF1-α R* | ACTGGAGTGCTTAAACCTGG |
